# Inhibition of human cytochromes P450 2A6 and 2A13 by flavonoids, acetylenic thiophenes and sesquiterpene lactones from *Pluchea indica* and *Vernonia cinerea*

**DOI:** 10.1080/14756366.2017.1363741

**Published:** 2017-08-31

**Authors:** Supattra Boonruang, Khanistha Prakobsri, Phisit Pouyfung, Ekaruth Srisook, Aruna Prasopthum, Pornpimol Rongnoparut, Songklod Sarapusit

**Affiliations:** a Bioengineering Program, Faculty of Engineering, Burapha University, Muang, Chonburi, Thailand;; b Department of Biochemistry, Faculty of Science, Mahidol University, Ratchathewi, Bangkok, Thailand;; c Department of Chemistry and Center for Innovation in Chemistry, Faculty of Science, Burapha University, Muang, Chonburi, Thailand;; d Department of Biochemistry and Center for Innovation in Chemistry, Faculty of Science, Burapha University, Muang, Chonburi, Thailand

**Keywords:** CYP2A6, CYP2A13, acetylenic thiophenes, sesquiterpene lactones, flavonoids

## Abstract

The human liver cytochrome P450 (CYP) 2A6 and the respiratory CYP2A13 enzymes play role in nicotine metabolism and activation of tobacco-specific nitrosamine carcinogens. Inhibition of both enzymes could offer a strategy for smoking abstinence and decreased risks of respiratory diseases and lung cancer. In this study, activity-guided isolation identified four flavonoids **1**–**4** (apigenin, luteolin, chrysoeriol, quercetin) from *Vernonia cinerea* and *Pluchea indica,* four hirsutinolide-type sesquiterpene lactones **5**–**8** from *V. cinerea*, and acetylenic thiophenes **9**–**11** from *P. indica* that inhibited CYP2A6- and CYP2A13-mediated coumarin 7-hydroxylation. Flavonoids were most effective in inhibition against CYP2A6 and CYP2A13, followed by thiophenes, and hirsutinolides. Hirsutinolides and thiophenes exhibited mechanism-based inhibition and in irreversible mode against both enzymes. The inactivation kinetic *K*_I_ values of hirsutinolides against CYP2A6 and CYP2A13 were 5.32–15.4 and 0.92–8.67 µM, respectively, while those of thiophenes were 0.11–1.01 and 0.67–0.97 µM, respectively.

## Introduction

Tobacco smoking can be the main cause of adverse human health effects and many tobacco-related diseases, including respiratory diseases and lung cancer, which is a leading cause of cancer death worldwide. The use of tobacco products continues to be widespread and might be responsible for approximately six million deaths in the world each year, as estimated by the WHO^1^. Nicotine is the main addictive constituent in tobacco, and tobacco-specific procarcinogenic nitrosamines are also found in tobacco and tobacco smoke. These compounds, including N-nitrosonornicotine (NNN) and 4-(methylnitrosamino)-1-(3-pyridyl)-1-butanone (NNK), have been recognised being associated with oesophagus cancer and lung cancer risks, respectively^2^.

In human, the liver heme-containing cytochrome P450 (CYP) 2A6 and the respiratory tract-expressed CYP2A13 play role in nicotine metabolism and they mediate metabolic activation of NNK to exhibit carcinogenic potential^3^^,^^4^. Addiction to nicotine could also cause prolonged exposure to these deleterious carcinogens in tobacco and tobacco smoke, particularly via CYP2As-mediated activation and might result in tumorigenesis risk^5^. Polymorphic variations of CYP2A6 gene conferring individual differences in the rate of nicotine metabolism are reported correlation to smoking rates^6^. An epidemiological association study indicates that human CYP2A6 and CYP2A13 genotypes with reduced enzyme activity may be related to a decreased cancer risks^7^^,^^8^, possibly due to the decreased CYP2As-mediated nitrosamine activation. Moreover, there was a link between CYP2A13 alleles and individual susceptibility to early onset of lung cancer in women^9^ and transgenic CYP2A13 could mediate NNK-induced lung tumorigenesis in a CYP2A13-humanised mouse model^5^. Thus, inhibition of the human CYP2As enzymes might promote smoking abstinence and reduce exposure to the carcinogenic NNK and hence decrease risks of carcinogenesis development. Inhibition of nicotine metabolisms mediated by human CYP2A6 by methoxsalen, a CYP2As inhibitor, has resulted in the inhibition of first-pass nicotine metabolism and combination of treatment of nicotine with methoxsalen could decrease the extent of smoking in smokers^10^. In this context, natural plant products could be a potential source of phytochemical inhibitors of CYP2A6 and CYP2A13 enzymes, as plant compounds are considered safe and easily biodegradable.

We previously reported inhibition effects of flavonoids and sesquiterpene lactones isolated from *Vernonia cinerea* against CYP2A6 and monoamine oxidases that are therapeutic targets for the abstinence of nicotine addiction^11^. The results supported traditional medicine use of *V. cinerea* for smoking cessation, possibly via maintaining blood nicotine and dopamine levels^11^. *V. cinerea* (Asteraceae) is a perennial herbaceous medicinal plant in the Asteraceae family and is found distributed in Asia, India and Africa. It has been used for various remedies, such as fever, cold, sore throat, malaria, asthma, bronchitis^12^, and has shown inhibiting human lung tumour formation and consisting of smoking abstinence effects in volunteer smokers^13^^,^^14^. Whether *V. cinerea* comprises of compounds that exhibit inhibitory effects against CYP2A13 has not been explored and it should be beneficial to gain an understanding of inhibitory roles of *V. cinerea* compounds against both CYP2A6 and CYP2A13 enzymes. Moreover, our prior search for naturally occurring plant compounds that could inhibit CYP2As has revealed that *Pluchea indica* possesses promising inhibitory effects against CYP2As enzymes. *P. indica* (L.) Less (Asteraceae) is a shrub found widely distributed in Asia. It has been used in traditional medicine for treating respiratory diseases, including sore throat and tuberculosis, and its leaves have been reported pharmacological activities such as antituberculosis, anti-inflammatory and antiproliferative effects on nasopharyngeal cancer cells^15–17^.

**Figure 1.**
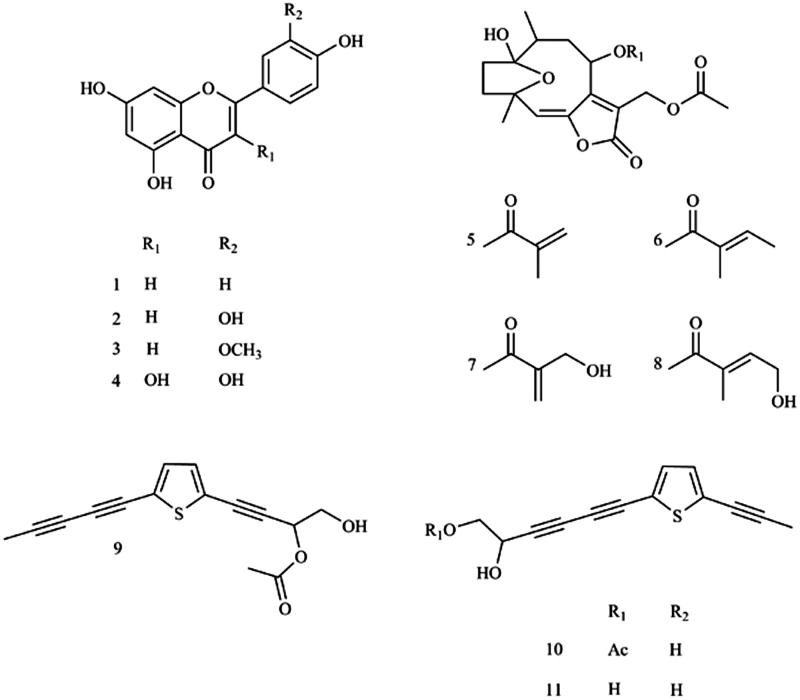
Chemical structures of apigenin (**1**), luteolin (**2**), chrysoeriol (**3**), quercetin (**4**), 8α-(2-methylacryloyloxy)-hirsutinolide-13-*O*-acetate (**5**), 8α-tigloyloxyhirsutinolide-13-*O*-acetate (**6**), 8α-(4-hydroxymethacryloyloxy)-hirsutinolide-13-*O*-acetate (**7**), 8α-(4-hydroxytigloyloxy)-hirsutinolide-13-*O*-acetate (**8**), 2-(penta-1,3-diyn-1-yl)-5–(4-acetoxy-3-hydroxybuta-1-yn-1-yl) thiophene (**9**), 2-(prop-1-inyl)-5–(6-acetoxy-5-hydroxyhexa-1, 3-diinyl) thiophene (**10**), 2-(prop-1-inyl)-5–(5, 6-dihydroxyhexa-1,3-diinyl) thiophene (**11**). Chemical structure was produced using ChemDraw Professional 8.

In this study, four flavonoids (apigenin (**1**), luteolin (**2**), quercetin (**4**), from both *V. cinerea* and *P. indica* and chrysoeriol (**3**) from *V. cinerea*), four hirsutinolide-type sesquiterpene lactones **5**–**8** from *V. cinerea*, and three acetylenic thiophenes **9**–**11** from *P. indica* (see chemical structure in Figure 1) were obtained by activity-guided isolation. We investigated inhibition effects of thiophenes **9**–**11** against coumarin 7-hydroxylation mediated by CYP2As enzymes, using coumarin as a probe substrate of both CYP2A6 and CYP2A13 enzymes, while inhibition of flavonoids **1**–**4** and hirsutinolides **5**–**8** was investigated toward CYP2A13 and compared to previous results of CYP2A6 (11). Kinetics and modes of inhibition of purified compounds were determined. Mechanism-based inhibition presented by concentration-, time- and NADPH-dependent inhibition of compounds was also examined. Investigation of the three different chemical structural types of compounds might also give insights into the nature of inhibitory compounds against human CYP2As enzymes.

## Materials and methods

### Chemicals

Nicotinamide adenosine diphosphate reduced form (NADPH), methoxsalen, (*S*)-nicotine, glutathione (GSH), catalase, semicarbazide, potassium ferricyanide and coumarin were purchased from Sigma-Aldrich (St. Louis, MO). Silica gel (230–400 mesh), pre-coated Kiesel gel 60 F254 thin-layer chromatography (TLC) sheets and Sephadex LH-20 gel were obtained from Merck (Billerica, MA). AR-grade ethanol (EtOH), methanol, *n*-hexane, ethyl acetate (EtOAc), diethyl ether and HPLC-grade acetonitrile (ACN) were supplied by RCI Labscan (Bangkok, Thailand).

### Isolation of compounds from V. Cinerea

Aerial parts of *V. cinerea* were obtained from Thai traditional medicine market and plant materials were identified as previously described (voucher sample number 7803, 11). The EtOH crude extract and partitioned hexane and EtOAc extracts of *V. cinerea* were prepared and subjected to *in vitro* inhibition assay using 10 µg/ml each as described^11^. As reported, the hexane fraction comprised largely chlorophylls that inhibited CYP2A6 (11), and in this study, inhibition effect on CYP2A13 of the hexane fraction was also, in majority, attributed to chlorophylls. For EtOAc extract, identification of the active inhibitory components using inhibition-guided isolation was achieved by subjection of the extract to silica gel column chromatography, successive elution with diethyl ether: EtOAc (100:0 to 0:100), pooled upon TLC analysis to afford 12 fractions. Those with at least 50% inhibition effect were chosen for compound purification by HPLC analyses using conditions previously described^11^. Three flavonoids that elicited inhibitory activity were obtained from fractions 5 (apigenin, **1**) and 6 (luteolin, **2** and chrysoeriol, **3**). Fractions 7–9 (diethyl ether: EtOAc =30–60: 70–40) were combined and further purified using silica gel chromatography to yield seven subfractions, followed by sephadex LH-20 gel chromatography and HPLC using conditions previously described^11^. We found one flavonoid (quercetin (**4**) in subfraction 7.7) and four hirsutinolide-type sesquiterpene lactones (8α-(2-methylacryloyloxy)-hirsutinolide-13-O-acetate (**5**), 8α-tigloyloxyhirsutinolide-13-O-acetate (**6**) in subfraction 7.4, 8α-(4-hydroxymethacryloyloxy)-hirsutinolide-13-O-acetate (**7**) and 8α-(4-hydroxytigloyloxy)-hirsutinolide-13-O-acetate (**8**) in subfraction 7.5) comprised inhibitory activity against CYP2A13. These compounds also previously exhibited inhibition effect on CYP2A6 enzyme^11^. The yields of these compounds and determination of chemical structures of these flavonoids and sesquitetpene lactones were as described^11^. Chemical structures of compounds isolated in this study are shown in Figure 1.

### Isolation of compounds from P. Indica

The aerial parts of *P. indica* were purchased from the Community Enterprise, Bon subdistrict, Khlung, Chantaburi province, Thailand and identified by Dr. Benchawan Chewprecha, Department of Biology, Faculty of Science, Burapha University, Thailand. A voucher specimen (BCSK-003) was deposited at the Faculty of Science, Burapha University. The dried and ground material (1 kg) was macerated in 95% EtOH three times at room temperature to yield 60 g of EtOH extract and partitioned with hexane and EtOAc, yielding hexane (17 g), EtOAc (15 g) and aqueous (28 g) soluble extracts.

The active components in EtOAc extract were separated by silica gel column chromatography, followed by successive elution with EtOAc: diethyl ether (0:100 to 100:0), and a final elution with methanol to afford eight fractions. Fractions 4 (1.5 g) and 5 (0.9 g) containing at least 50% inhibition effects on both enzymes at 10 µg/ml were subjected to HPLC analysis, using semipreparative RP-18 column (47.8 mm ×100 mm, Water Corporation, Ireland). The solvent system-contained ACN and water and was set as follows: 0 min, 30% v/v ACN in water; 0–5 min, a linear gradient of 30–40% ACN; 5–10 min, 40–50% ACN; 10–15 min, 50% ACN; 15–20 min, 50–100% ACN; 20–25 min, 100% ACN; 25–30 min, cycled back to 30% ACN, with total running time of 35 min and flow rate at 1.5 ml/min. Three flavonoids eluted at 8.7, 9.7, 11.48 min were identified as quercetin (**4**, 5.5 mg), luteolin (**2**, 1.45 mg) and apigenin (**1**, 1.45 mg), respectively.

Hexane extract was subjected to silica gel column chromatography and successively eluted with a solvent system containing hexane: EtOAc (100:0 to 0:100), and a final elution was done with methanol to resolve seven fractions. The yields obtained from fractions 1–7 were 0.38, 2.55, 4.25, 6.39, 1.75, 1.19 and 0.45 g, respectively. Fractions 2–4 were further separated by sephadex LH-20 gel column chromatography and eluted with chloroform: methanol (2:1) to afford five fractions. Fractions 2 (6.46 mg), 4 (8.84 mg) and 5 (15.79 mg) containing inhibitory activity toward both enzymes were proceeded to the final purification step by HPLC, with stepwise elution utilising solvent system containing ACN and water (flow rate at 1.5 ml/min) that was set as follows: 0 min, 25% v/v ACN/water, a linear gradient of 25–100% ACN; 0–20 min,100% ACN; 25–30 min, 100–25% ACN; 30–35 min, a final equilibration at 25% ACN and with a total running time of 35 min. The compound in fraction 2 eluted at 22.0 min on HPLC was identified as 2-(penta-1,3-diyn-1-yl)-5-(4-acetoxy-3-hydroxybuta-1-yn-1-yl) thiophene (**9**, 6.46 mg), compounds in fractions 4 and 5 that were eluted at 16.36 min and 13.1 min were 2-(prop-1-inyl)-5-(6-acetoxy-5-hydroxyhexa-1, 3-diinyl) thiophene (**10**, 8.84 mg) and 2-(prop-1-inyl)-5-(5, 6-dihydroxyhexa-1,3-diinyl) thiophene (**11**, 15.79 mg), respectively. The chemical structures of compounds **9**–**11** were elucidated based on UV spectra determined on HPLC, electrospray ionisation source equipped with tandem mass spectrometer (ESI-MS/MS) and nuclear magnetic resonance (Bruker Daltonics GmbH, Bremen, Germany) spectra analysis, compared to those of previous reports^18^^,^^19^.

### Inhibition assay

Inhibitory effect of plant extracts, fractions, subfractions and constituents was determined by inhibition assay on CYP2A6- and CYP2A13-mediated coumarin 7-hydroxylation. The recombinant human CYP2A6, CYP2A13 and rat NADPH-dependent cytochrome P450 oxidoreductase (CYPOR) proteins were expressed, purified, according to the methods previously described^20^. Enzymatic reconstitution assay, containing CYP2A, CYPOR as a redox partner enzyme and coumarin probe substrate (2 µM, close to *K*_m_ value), was performed at room temperature as described^20^. Rate of 7-hydroxycoumarin product formation was measured on spectrofluorometer (Shimadzu, Kyoto, Japan) at λ_ex_ = 355 nm, λ_em_ = 460 nm. Inhibition effects were determined by incubation with extracts, fractions or purified compounds with coumarin substrate in the reaction mixture and calculated as per cent relative inhibition compared with vehicle control reaction^20^ and IC_50_ values were calculated using GraphPad Prism 5 (GraphPad Software Inc., La Jolla, CA). 

Mode of inhibition and inhibition kinetics (*K*_i_) were determined by performing inhibition with coumarin substrate (0–40 µM) and with different concentrations of each test compound (0–100 µM). Compounds that primarily showed an increased inhibition under 10-min pre-incubation condition in the presence of NADPH compared to the absence of NADPH were further explored for time- and NADPH-dependent inhibition that defined a mechanism-based inhibition (MBI). MBI requires NADPH and time to generate reactive intermediate as inhibitor and ultimately inactivates the enzyme. MBI was evaluated by pre-incubation with each test inhibitor in the reaction at different time periods (0, 10, 20 and 30 min) in the presence of NADPH for time-dependent assay, as described^20^. The apparent inactivation constants, *K*_I_ and *K*_inact_, were obtained from double reciprocal plots of *k*_obs_ versus inhibitor concentrations by linear regression analysis on GraphPad Prism 5. Methoxsalen was used as a control mechanism-based inhibitor for time- and NADPH-dependent inhibition assays. Effects of trapping agents (2 mM GSH, 5 mM semicarbazide and 2000 U catalase), 70 µM potassium ferricyanide and dialysis were performed as previously described with compounds that exhibited MBI pattern^20^.

### Statistical analysis

Data shown in Tables 1 and 2 are expressed as means ± SDs of triplicate experiments. Comparison of data in Tables 1–3 was performed using Student’s *t*-test (Statistix 8.0, Analytical Software, Inc., Tallahassee, FL). Results with *p* ≤ 0.05 were considered being significantly different.

**Table 1.** IC_50_ values of extracts and compounds from *V. cinerea* and *P. indica* against CYP2A6 and CYP2A13

	CYP2A6^a^		CYP2A13^a^	
Samples	Coincubation	Pre-incubation	Coincubation	Pre-incubation
*V. cinerea*				
Ethanol (µg/ml)	4.00 ± 0.14^b^^,^^c^	2.32 ± 0.01^b^^,^^c^	3.57 ± 0.008^b^	2.54 ± 0.06^b^
Hexane (µg/ml)	3.31 ± 0.24^b^^,^^c^	1.65 ± 0.05^b^^,^^c^	3.25 ± 0.22^b^	2.03 ± 0.05^b^
Ethyl acetate (µg/ml)	2.90 ± 0.16^b^^,^^c^	1.57 ± 0.03^b^^,^^c^	2.28 ± 0.09	2.07 ± 0.08
Aqueous fraction (µg/ml)	>100	ND	>100	ND
Apigenin **1** (µM)	0.90 ± 0.07^c^	0.77 ± 0.16^c^	0.05 ± 0.01	0.04 ± 0.01
Luteolin **2** (µM)	1.38 ± 0.18^c^	1.26 ± 0.07^c^	0.18 ± 0.02	0.17 ± 0.01
Chrysoeriol **3** (µM)	1.14 ± 0.10^c^	0.99 ± 0.12^c^	0.82 ± 0.05	0.79 ± 0.01
Quercetin **4** (µM)	2.66 ± 0.24^c^	2.15 ± 0.38^c^	0.80 ± 0.01	0.74 ± 0.02
Hirsutinolide **5** (µM)	22.3 ± 2.5^b^^,^^c^	8.64 ± 0.37^b^^,^^c^	16.44 ± 3.10^b^	4.50 ± 0.18^b^
Hirsutinolide **6** (µM)	37.8 ± 3.5^b^^,^^c^	20.80 ± 1.10^b^^,^^c^	20.86 ± 1.46^b^	7.66 ± 0.98^b^
Hirsutinolide **7** (µM)	32.7 ± 2.2^b^^,^^c^	6.80 ± 0.77^b^^,^^c^	23.26 ± 1.35^b^	10.52 ± 0.49^b^
Hirsutinolide **8** (µM)	64.5 ± 5.8^b^^,^^c^	13.10 ± 2.40^b^^,^^c^	36.11 ± 1.39^b^	15.94 ± 0.67^b^
*P. indica*				
Ethanol (µg/ml)	8.32 ± 0.07^b^	4.73 ± 0.70^b^	8.37 ± 0.79^b^	5.44 ± 0.20^b^
Hexane (µg/ml)	8.38 ± 1.10^b^	3.34 ± 0.17^b^	5.31 ± 0.62^b^	3.39 ± 0.35^b^
Ethyl acetate (µg/ml)	3.52 ± 0.85	2.91 ± 0.09	5.15 ± 0.36	4.96 ± 0.10
Aqueous fraction (µg/ml)	>100	ND	90.0 ± 0.08	85.15 ± 0.11
Thiophene **9** (µM)	6.43 ± 1.29^b^	2.12 ± 0.19^b^	6.18 ± 0.28^b^	2.29 ± 0.34^b^
Thiophene **10** (µM)	4.44 ± 0.14^b^	2.97 ± 0.01^b^	2.94 ± 0.01^b^	1.15 ± 0.88^b^
Thiophene **11** (µM)	3.90 ± 0.20^b^	0.18 ± 0.01^b^	2.40 ± 0.33^b^	1.47 ± 0.12^b^

a Each value represents mean ± SD of triplicate experiments.

b Significant difference (*p* < .05) between IC_50_ values of coincubation versus pre-incubation.

c Data obtained from Prasopthum et al., 2015.

ND: not determined.

**Table 2.** Kinetics values and mode of inhibition of purified compounds from *V. cinerea* and *P. indica* against CYP2A6 and CYP2A13.

	CYP2A6^a^				CYP2A13^a^			
Compounds	*K*_i_ (µM)	*K*_I_ (µM)	*K*_inact_	Mode	*K*_i_ (µM)	*K*_I_ (µM)	*K*_inact_	Mode
*V. cinerea* (µM)								
Apigenin **1**	0.43 ± 0.17^b^	NA^b^	NA^b^	Mixed type^b^	0.014 ± 0.002	NA	NA	Mixed type
Luteolin **2**	0.80 ± 0.06^b^	NA^b^	NA^b^	Competitive^b^	0.07 ± 0.01	NA	NA	Competitive
Chrysoeriol **3**	0.63 ± 0.12^b^	NA^b^	NA^b^	Competitive^b^	0.28 ± 0.01	NA	NA	Competitive
Quercetin **4**	1.19 ± 0.27^b^	NA^b^	NA^b^	Competitive^b^	0.11 ± 0.01	NA	NA	Competitive
Hirsutinolide **5**	15.1 ± 2.1^b^	7.45 ± 0.62^b^	0.05 ± 0.01^b^	Mixed type^b^	5.61 ± 0.99	0.92 ± 0.17	0.03 ± 0.01	Mixed type
Hirsutinolide **6**	30.6 ± 1.5^b^	15.4 ± 1.80^b^	0.03 ± 0.01^b^	Mixed type^b^	7.97 ± 0.78	3.60 ± 1.05	0.06 ± 0.01	Mixed type
Hirsutinolide **7**	20.4 ± 5.4^b^	5.32 ± 0.29^b^	0.03 ± 0.01^b^	Mixed type^b^	12.56 ± 1.26	8.67 ± 0.15	0.06 ± 0.01	Mixed type
Hirsutinolide **8**	42.3 ± 8.4^b^	7.64 ± 1.24^b^	0.09 ± 0.01^b^	Mixed type^b^	15.76 ± 2.5	8.62 ± 0.37	0.10 ± 0.01	Mixed type
*P. indica* (µM)								
Thiophene **9**	3.23 ± 0.43	0.88 ± 0.09	0.10 ± 0.02	Mixed type	2.96 ± 0.10	0.97 ± 0.04	0.08 ± 0.01	Mixed type
Thiophene **10**	2.07 ± 0.23	1.01 ± 0.07	0.11 ± 0.01	Mixed type	1.12 ± 0.09	0.86 ± 0.09	0.06 ± 0.01	Mixed type
Thiophene **11**	1.80 ± 0.18	0.11 ± 0.02	0.10 ± 0.02	Mixed type	1.08 ± 0.05	0.67 ± 0.08	0.05 ± 0.01	Mixed type

a Each value represents mean ± SD of triplicate experiments.

b Data obtained from Prasopthum et al., 2015.

NA: not applicable.

**Table 3.** Effects of various trapping agents and dialysis of purified compounds from *V. cinerea* and *P. indica* on CYP2A6 and CYP2A13

	Per cent remaining activity^a^						
	Pre-incubation	Dialysis (−) NADPH	Dialysis (+) NADPH	5 mM semicarbazide	2 mM GSH	2000 U catalase	70 μM K_3_Fe(CN)_6_
CYP2A6							
*P. indica*							
5 µM Thiophene **9**	30.14 ± 3.70	89.33 ± 7.00	20.09 ± 5.21	26.75 ± 2.39	21.63 ± 1.97	26.89 ± 3.07	22.71 ± 1.58
5 µM Thiophene **10**	35.22 ± 3.22	87.48 ± 9.25	23.69 ± 2.06	21.12 ± 3.31	28.52 ± 1.72	24.09 ± 4.49	19.18 ± 1.83
5 µM Thiophene **11**	12.54 ± 1.93	82.39 ± 2.49	10.08 ± 1.84	12.92 ± 3.47	8.42 ± 0.90	14.42 ± 1.41	11.23 ± 2.96
CYP2A13							
*V. cinerea*							
10 µM Hirsutinolide **5**	29.28 ± 2.53	86.03 ± 6.65	18.63 ± 0.94	24.35 ± 2.54	28.20 ± 6.54	20.27 ± 6.24	24.07 ± 0.62
10 µM Hirsutinolide **6**	28.35 ± 2.92	85.34 ± 4.98	22.77 ± 1.13	25.04 ± 3.81	33.60 ± 4.80	26.61 ± 3.86	25.91 ± 1.87
10 µM Hirsutinolide **7**	22.16 ± 1.88	93.27 ± 2.10	21.35 ± 4.45	26.60 ± 1.25	18.29 ± 1.54	20.90 ± 5.18	21.39 ± 2.75
20 µM Hirsutinolide **8**	23.89 ± 3.17	92.39 ± 4.03	18.37 ± 1.10	20.70 ± 2.92	25.79 ± 4.20	19.44 ± 1.39	21.82 ± 1.85
*P. indica*							
5 µM Thiophene **9**	28.97 ± 4.57	85.34 ± 4.98	20.92 ± 0.24	23.17 ± 3.38	23.17 ± 3.38	22.14 ± 2.39	24.86 ± 3.95
5 µM Thiophene **10**	28.95 ± 1.25	93.02 ± 6.58	21.43 ± 3.95	26.98 ± 8.11	26.98 ± 8.11	22.18 ± 2.38	26.89 ± 1.75
5 µM Thiophene **11**	20.17 ± 2.76	96.84 ± 0.73	19.63 ± 4.21	19.58 ± 2.73	23.48 ± 1.72	19.54 ± 2.95	18.87 ± 0.18

a Data represent means ± SD of triplicate experiments.

## Results and discussion

In this study, extracts and constituents of *V. cinerea* exhibiting inhibition against the recombinant CYP2A13 were examined using enzymatic inhibition assays and their IC_50_ values were compared to those previously reported for the recombinant human CYP2A6 (11). Extracts and constituents of *P. indica* were also investigated inhibition activities against CYP2A6 and CYP2A13 enzymes. Upon compound purification from both plant extracts using activity-guided isolation, we obtained compounds that could be grouped into flavonoids, hirsutinolide-type sesquiterpene lactones and acetylenic thiophenes. In *V. cinerea*, we found flavones **1**–**3** (apigenin, luteolin, chrysoeriol, respectively), one flavonol (quercetin, **4**) and hirsutinolides **5**–**8** as active components in inhibition against both enzymes, while flavonoids apigenin, luteolin, quercetin and acetylenic thiophenes **9**–**11** were found active in *P. indica*. With the purification conditions described, all active components in *V. cinerea* resided in the EtOAc extract and there was mostly chlorophyll found in hexane extract that elicited inhibition on CYP2A13. This was also previously found for the inhibition of CYP2A6 (11). The partially purified chlorophyl in *V. cinerea* hexane extract could inhibit CYP2A6 (the apparent IC_50_ value of 4.46 ± 0.16 µg/ml, 11) and CYP2A13 (IC_50_ value of 3.44 ± 0.24 µg/ml). In *P. indica*, flavonoids **1, 2, 4** were found in EtOAc extract and thiophenes **9**–**11** were in hexane extract.

The IC_50_ values, modes of inhibition and kinetic parameters were determined for each of the purified compounds and are displayed in Tables 1 and 2. The purified compounds **1**–**11** displayed higher inhibition effects on CYP2A13 than CYP2A6, especially flavonoids **1**–**4** possessed approximately 1.4- to 18-fold lower IC_50_ values against CYP2A13 than CYP2A6 (Table 1). These flavonoids potentially inhibited both enzymes in reversible mode (Table 2), with *K*_i_ values of 0.01–0.28 µM on CYP2A13 and 0.43–1.19 µM on CYP2A6. Thiophenes **9**–**11** inhibited both enzymes better than hirsutinolides **5**–**8** but inhibited to a lesser extent than flavonoids **1**–**4**.

Among flavonoids displayed in Table 1, the flavonol quercetin (**4**) was less active in inhibition on both enzymes than flavones apigenin (**1**) and luteolin (**2**). The methoxylated flavone, chrysoeriol (**3**), was similarly effective as the flavone **1**, **2** in inhibition of CYP2A6 but was poorer for CYP2A13 inhibition. Similarly, previous study observed that most flavones tested comprised high inhibitory activity towards CYP2A6 and CYP2A13, with lesser activity found for flavonols^21^.

**Figure 2.**
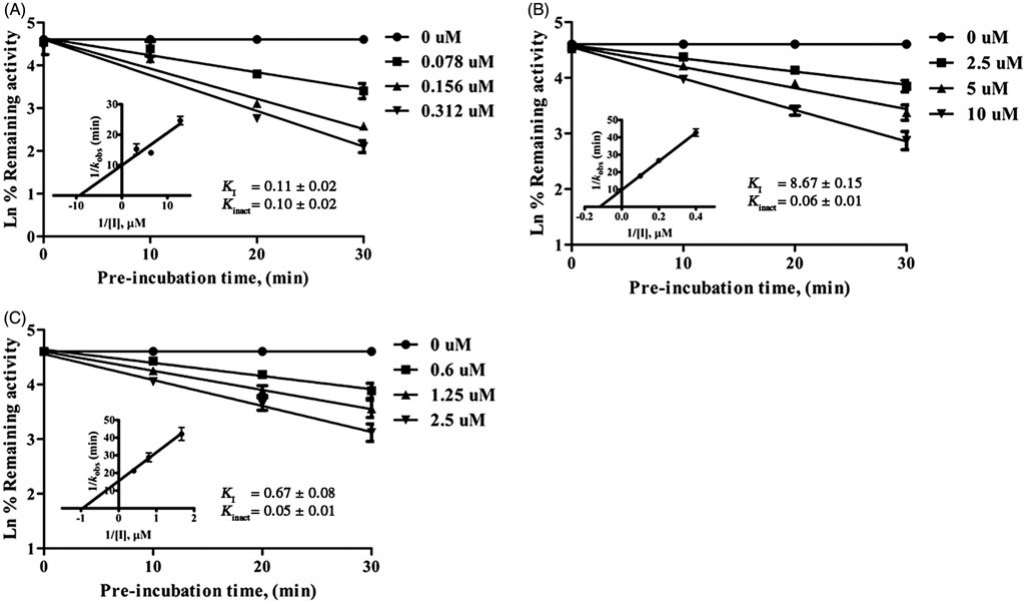
Time- and concentration-dependent inactivation and kinetics of inhibition of CYP2A6-mediated coumarin 7-hydroxylation by 2-(prop-1-inyl)-5–(5, 6-dihydroxyhexa-1,3-diinyl) thiophene **11** (A), and of CYP2A13-mediated coumarin 7-hydroxylation by 8α-(4-hydroxymethacryloyloxy)-hirsutinolide-13-*O*-acetate **7** (B), and 2-(prop-1-inyl)-5–(5, 6-dihydroxyhexa-1,3-diinyl) thiophene **11** (C). Data are represented as mean ± SD of triplicate experiments.

A 10-min pre-incubation with compounds **1**–**11** in the presence of NADPH to primarily test for time-dependent inhibition (TDI) indicated that hirsutinolides **5**–**8** and thiophenes **9**–**11** revealed an increased inhibition towards both enzymes compared to coincubation condition (Table 1). Compounds **5**–**11** further exhibited TDI and NADPH-dependent inhibition (unreported data), indicating that inhibition of hirsutinolides **5**–**8** was MBI towards CYP2A13, similar to the previous report on CYP2A6 (11), and thiophenes **9**–**11** were mechanism-based inhibitors of both enzymes (see examples of TDI of hirsutinolide **7** and thiophene **11** in Figure 2 and of remaining compounds in Supplemental Figure S1). The *K*_i_ values were determined for all purified compounds and showed that the values were in similar trend as their IC_50_ values against both enzymes (Table 2). The inactivation kinetics, *K*_I_ and *K*_inact_, obtained for hirsutinolides and thiophenes (Table 2) indicated that thiophenes **9**–**11** were more potent inactivators of CYP2A6 and CYP2A13 than hirsutinolides **5**–**8**. For thiophenes, it might be thought that it was the thiophene ring (Figure 1) that interacted with the CYP2As enzyme active sites to result in enzyme inactivation, as previously reported for CYPs^22^. On the other hand, the alkyne moiety that was attached to the thiophene ring of compounds **9**–**11** could cause efficient and irreversible inhibition, as compounds comprising of acetylene structures displayed MBI against the human CYP1A1/2 and CYP2B1/2 enzymes^23^.

To explore whether inactivation of CYP2As by thiophenes **9**–**11** and of CYP2A13 by hirsutinolides **5-8** could be reversed, the removal of reversible inhibitory compounds by dialysis and addition of trapping agents (GSH, semicarbazide, catalase or potassium ferricyanide) in the inhibition reaction mixture were performed. As shown in Table 3, neither dialysis (in the presence of NADPH) nor addition of trapping agents protected CYP2A13 from mechanism-based inactivation by hirsutinolides **5**–**8**, similar to effects previously observed on CYP2A6 (11). Similarly, mechanism-based inactivation of both CYP2A6 and CYP2A13 by thiophenes **9**–**11** could not be reversed (Table 3).

Comparing among the three groups of compound structure, judging from IC_50_ values, flavones ranked first in inhibition, followed by acetylenic thiophenes, while hirsutinolides were weakest inhibitors of CYP2As (Table 1). Similarly, as shown by *K*_i_ values, thiophenes exerted better binding on CYP2A6 and CYP2A13 than hirsutinolides but were slightly inferior to flavonoids. All compounds **1**–**11** inhibited both CYP2As in competitive or mixed-type modes and inhibited CYP2A13 with higher degree than CYP2A6 (Tables 1 and 2), possibly owing to the larger active site volume of CYP2A13 than CYP2A6^24^, and thus, CYP2A13 might better accommodate different inhibitory compounds in the active site. The irreversible inhibitors, acetylenic thiophenes **9**–**11** and hirsutinolide-type sesquiterpene lactones **5**–**8**, could be effective inhibitors of the human CYP2A6 and CYP2A13, as time should be required for the recovery of enzyme activity via *de novo* enzyme synthesis *in vivo*. The inactivation kinetic values (*K*_I_) for natural plant compounds have been reported, for example, the values of rhinacanthin-A, rhinacanthin-B, rhincanthin-C, decursinol angelate and selegiline, were 0.69, 0.44, 0.97, 2.42 and 15.6 µM, respectively, against CYP2A6^20^^,^^25^^,^^26^. Moreover, *K*_I_ values for phenylpropyl isothiocyanate, rhinacanthin-A, rhinacanthin-B, rhinacanthin-C, phenylhexyl isothiocyanate and tert-butyl isothiocyanate against CYP2A13 were 0.14, 0.42, 0.16, 1.68, 1.1 and 4.3 µM, respectively^20^^,^^27^. Thus, comparing to natural occurring plant compounds that were found irreversible inhibitors towards CYP2A6 and CYP2A13, the acetylenic thiophenes **9**–**11** (*K*_I_ values of 0.11–1.01 µM against CYP2A6 and 0.67–0.97 µM against CYP2A13) could bind CYP2As comparable to rhinacanthin-B and rhinacanthin-C and were more potent than decursinol angelate and selegiline. Moreover, the major flavonoid luteolin, hirsutinolide **6** (in *V. cinerea*) and thiophene **11** (in *P. indica*) showed inhibition on CYP2A6- and CYP2A13-mediated nicotine metabolisms (11 and Supplemental Figure S2), implicating that the active compounds **1**–**11** in both plants might also inhibit nicotine metabolism catalysed by both enzymes. Furthermore, the methoxylated flavone chrysoeriol **3** expressed potent inhibition on both CYP2As and MAO-B, with *K*_i_ values within 1 µM (11 and results reported herein), and thiophenes **9**–**11** were irreversible inhibitors of both CYP2As. These compounds might possibly serve as potential lead compounds for the development of chemopreventive agents for cigarette smokers. However, whether they could selectively inhibit CYP2A6 and CYP2A13 requires further investigation. It has been reported that chronically exposure to nicotine could cause proinflammatory effect on neutrophils and oxidative damage in lung of the experimental rat^28^, administration of *P. indica* and *V. cinerea* might also be effective in the alleviation of oxidative damage in lung caused by nicotine exposure as they have been reported comprising anti-oxidative and anti-inflammatory activities^12^^,^^16^. Taken together, these inhibitory compounds isolated from *V. cinerea* and *P. indica* and the composite of these compounds or extracts of these two medicinal plants might have implications for smoking cessation and reduced risks of respiratory diseases and lung cancer.

## Supplementary Material

IENZ_1363741_Supplementary_Material.pdf
