# Supplementary material for: Inhibition of human cytochromes P450 2A6 and 2A13 by flavonoids, acetylenic thiophenes and sesquiterpene lactones from Pluchea indica and Vernonia cinerea
Source: J Enzyme Inhib Med Chem. 2017 Aug 31;32(1):1136–42. doi: 10.1080/14756366.2017.1363741 (PMC6009911; doi:10.1080/14756366.2017.1363741)
Supplement: IENZ_1363741_Supplementary_Material.pdf [file IENZ_A_1363741_SM7455.pdf]

SUPPORTING INFORMATION

Inhibition of human cytochromes P450 2A6 and 2A13 by flavonoids, acetylenic thiophenes and sesquiterpene lactones from *Pluchea indica* and *Vernonia cinerea*

Supattra Boonruang[1,*] , Khanistha Prakobsri[1, *], Phisit Pouyfung[2], Ekaruth Srisook[3], Aruna Prasopthum[2], Pornpimol Rongnoparut[2], and Songklod Sarapusit[4,**]

[1]*Bioengineering Program, Faculty of Engineering, Burapha University, 169 Long-Hard Bangsaen Rd., Muang, Chonburi 20131, Thailand.*

[2]*Department of Biochemistry, Faculty of Science, Mahidol University, 272 Rama 6 Rd., Ratchathewi, Bangkok 10400, Thailand*

[3]*Department of Chemistry and Center for Innovation in Chemistry, Faculty of Science, Burapha University, 169 Long-Hard Bangsaen Rd., Muang, Chonburi 20131, Thailand.*

[4]*Department of Biochemistry and Center for Innovation in Chemistry, Faculty of Science, Burapha University, 169 Long-Hard Bangsaen Rd., Muang, Chonburi 20131, Thailand.*

[*] These authors contributed equally to this work

**Correspondence to:

Songklod Sarapusit, Ph.D.

Department of Biochemistry, Faculty of Science, Burapha University, 169 Long-Hard Bangsaen Rd., Muang, Chonburi 20131, Thailand.

Tel 66-3058-3810; FAX 66-3495-3839

E-mail: songklod@go.buu.ac.th

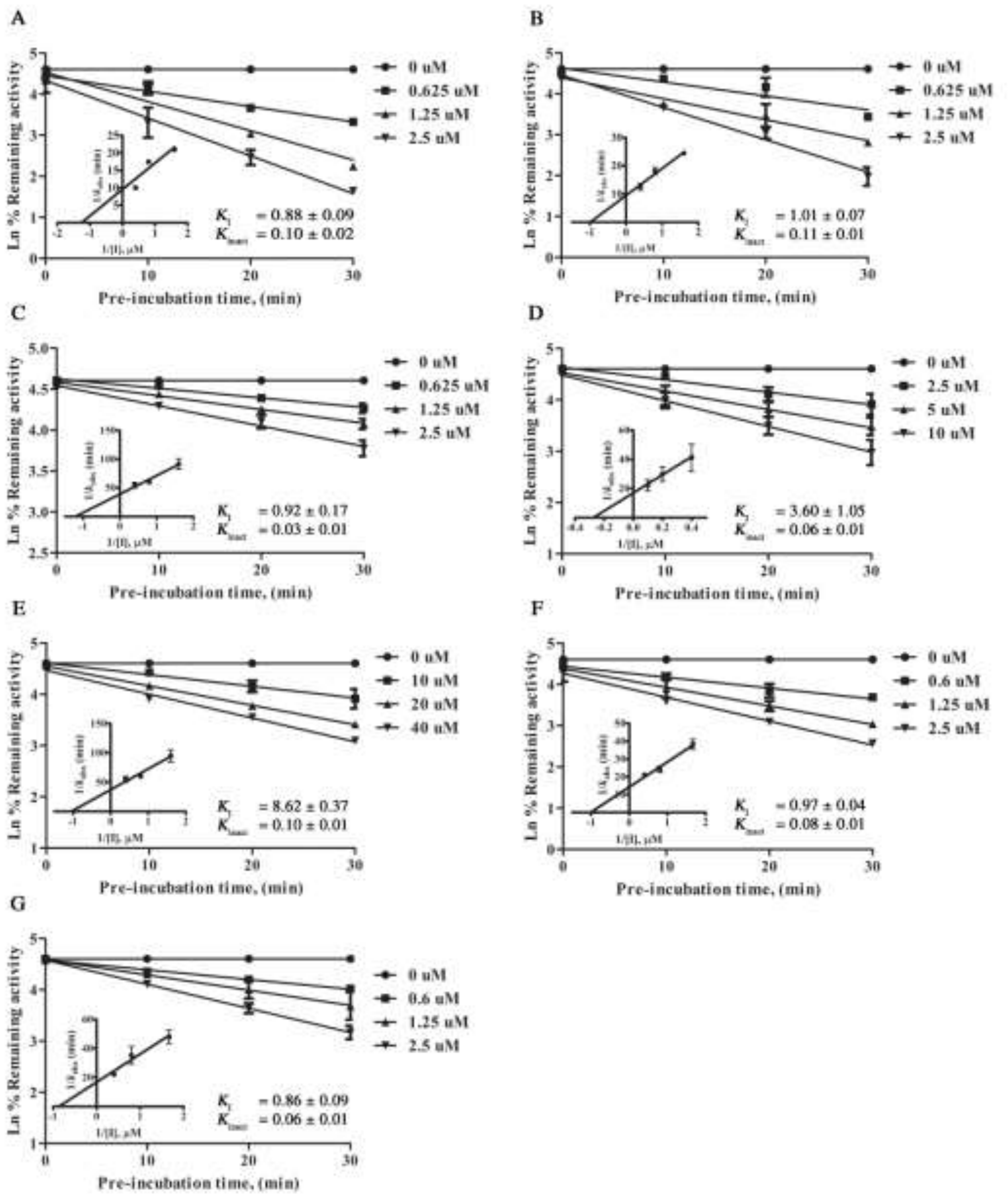

Supplementary Figure S1. Time- and concentration-dependent inactivation and kinetics of inhibition of CYP2A6-mediated coumarin 7-hydroxylation by 2-(penta-1,3-diyn-1-yl)-5-(4-acetoxy-3-hydroxybuta-1-yn-1-yl) thiophene **9** (A), 2-(prop-1-inyl)-5-(6-acetoxy-5-hydroxyhexa-1, 3-diinyl) thiophene **10** (B), and of CYP2A13-mediated coumarin 7-hydroxylation by 8α-(2-methylacryloyloxy)-hirsutinolide-13-*O*-acetate **5** (C), 8α-tigloyloxyhirsutinolide-13-*O*-acetate **6** (D), 8α-(4-hydroxytigloyloxy)-hirsutinolide-13-*O*-acetate **8** (E), and 2-(penta-1,3-diyn-1-yl)-5-(4-acetoxy-3-hydroxybuta-1-yn-1-yl) thiophene **9** (F), and 2-(prop-1-inyl)-5-(6-acetoxy-5-hydroxyhexa-1, 3-diinyl) thiophene **10** (G). Data are represented as mean ± SD of triplicate experiments.

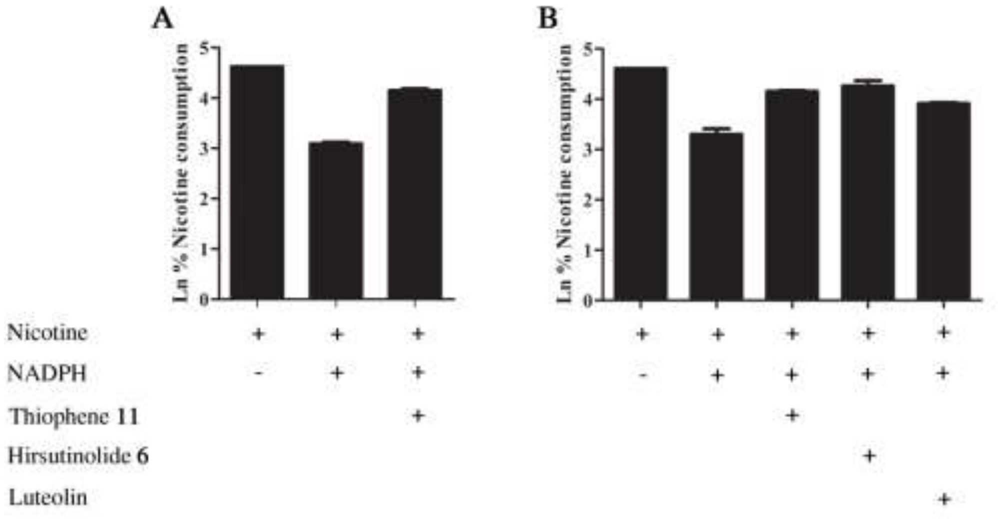

Supplementary Figure S2. Inhibition effects of thiophene **11** on CYP2A6-mediated nicotine metabolism (A), and of thiophene **11**, luteolin, hirsutinolide **6** on CYP2A13-mediated nicotine metabolism (B). Nicotine metabolism in the presence and absence of inhibitory test compounds, at IC_{50} value, was performed by incubation of the reaction mixture with nicotine substrate (a final concentration of 50 μM) prior to initiation of each reaction with 50 μM NADPH at room temperature. Each reaction was terminated by addition of ice-cold acetronitrile at 30 min incubation and 50 pmol 7-hydroxycoumarin as internal standard was added. The reaction was centrifuged (12,000 rpm, 10 min, 4°C) and 20 μl of each sample was analyzed on symmetry RP-C18 column (3.9×150 mm). Gradient condition was isocratic of 12% ACN: 2-propanol (7:3) in 10 mM potassium phosphate buffer (pH 7.4) at flow rate of 1 ml/min. The nicotine peak area in each reaction was compared with that of time zero. The enzyme activity was determined as percent substrate consumption over 30 min. The reactions were performed in duplicates.
